# Super-enhancer inhibitors THZ2 and JQ1 reverse temozolomide resistance in glioblastoma by suppressing SE-driven SOX9 expression

**DOI:** 10.20517/cdr.2025.105

**Published:** 2025-07-22

**Authors:** Xinqi Teng, Yiming Wang, Qiang Qu, Weixin Xu, Haihui Zhuang, Yiwen Wei, Yinghuan Dai, Jian Qu

**Affiliations:** ^1^Department of Pharmacy, the Second Xiangya Hospital, Central South University; Institute of Clinical Pharmacy, Central South University, Changsha 410011, Hunan, China.; ^2^Department of Pharmacy, Wuhan No.1 Hospital (Wuhan Integrated TCM & Western Medicine Hospital), Wuhan 430022, Hubei, China.; ^3^Department of Pharmacy, Xiangya Hospital, Central South University, Changsha 410078, Hunan, China.; ^4^National Clinical Research Center for Geriatric Disorders, Xiangya Hospital, Central South University, Changsha 410078, Hunan, China.; ^5^Department of Pathology, the Second Xiangya Hospital, Central South University, Changsha 410011, Hunan, China.; ^6^Hunan key laboratory of the research and development of novel pharmaceutical preparations, Changsha Medical University, Changsha 410219, Hunan, China.; ^#^Authors contributed equally.

**Keywords:** Glioblastoma, chemoresistance, super-enhancer, CDK7 inhibitor, SOX9

## Abstract

**Aim:** Glioblastoma (GBM) is the most malignant grade of glioma, characterized by high recurrence, poor prognosis, and frequent chemoresistance. There is an urgent need for alternative treatment strategies. In this study, we evaluated the effects of THZ2, a covalent inhibitor targeting the super-enhancer (SE) component CDK7, on GBM growth and chemoresistance. We also used another SE inhibitor, JQ1, to further validate the inhibitory effects of targeting SEs in GBM, thereby providing new treatment strategies for patients.

**Methods:** A variety of *in vitro* and *in vivo* assays were performed to explore the anti-GBM effects of SE inhibitors. We assessed the effects of SE inhibitors in combination with temozolomide (TMZ) on GBM cells and calculated the combination index. Additionally, CUT&RUN assays were conducted to examine protein-DNA interactions.

**Results:** THZ2 inhibited the proliferation, migration, and invasion of GBM cells and induced cell cycle arrest and apoptosis. Furthermore, both THZ2 and JQ1 exhibited synergistic antitumor effects when combined with TMZ in GBM cells. Notably, THZ2 reversed TMZ resistance in GBM cells by suppressing the expression of the SE-associated gene *SOX9*. We also found that *SOX9*, *CDK7*, and *BRD4* interact with histone H3K27ac.

**Conclusion:** Our findings demonstrate that SE inhibitors exert antitumor effects in GBM and act synergistically with TMZ. THZ2 may enhance chemosensitivity by downregulating the SE-related gene *SOX9*, and it holds promise as a novel therapeutic agent for GBM patients.

## INTRODUCTION

Gliomas are the most prevalent intrinsic malignancies of the central nervous system, with an estimated global incidence of approximately 3.62 cases per 100,000 individuals annually^[1,2]^. Among these, glioblastoma (GBM) is classified as the most aggressive form, representing grade IV malignant glioma. GBM exhibits near-universal therapeutic resistance, resulting in a high recurrence rate and poor prognosis, with a 5-year survival rate of only 6.9%^[1]^. The current standard treatment for GBM involves surgical resection followed by radiotherapy combined with concurrent and adjuvant temozolomide (TMZ) chemotherapy^[3,4]^. Despite these interventions, chemotherapy resistance and tumor relapse remain almost inevitable, leading to persistently low survival rates^[5]^. Therefore, there is an urgent need to advance the molecular understanding of GBM and to develop innovative therapeutic strategies against this lethal disease.

Recent investigations have highlighted the pivotal role of epigenetic mechanisms, including covalent DNA modifications, histone modifications, noncoding RNA regulation, and chromatin remodeling, in the malignant transformation of cancer cells^[6]^. Binding of transcription factors (TFs) to enhancers is an important initial step in gene expression^[7]^. Super-enhancers (SEs) are characterized by clusters of numerous TFs and cofactors, as well as high concentrations of mediator complexes, BRD4, CDK7, RNA polymerase II (RNAPII), and histone acetylation marks such as H3K27ac^[8,9]^. SEs are typically identified through ChIP-seq analysis of enhancer-associated TFs, cofactors (e.g., BRD4), or histone modifications (H3K27ac, H3K4me1)^[10]^. Increasing evidence indicates that SEs drive the pathological overexpression of key oncogenes in various cancers, thereby promoting tumor progression and therapeutic resistance^[11]^. As central regulatory hubs, SEs dictate cellular identity and fate, exerting master control over proliferation, differentiation, and oncogenic pathogenesis^[12-14]^. Our recent review also indicated that SEs play important roles in tumor chemoresistance^[15]^. SOX9 is a key master TF regulated by SEs and involved in their activation^[16,17]^. SOX9 expression is upregulated in various tumors and has been associated with TMZ resistance in gliomas^[18]^. However, the mechanism by which SEs regulate SOX9 to influence drug resistance in GBM cells remains unclear.

CDK7, a master regulatory cyclin-dependent kinase, plays a critical role in coordinating cell cycle progression and RNAPII-driven transcription^[19]^. Notably, SE-associated oncogenes are particularly vulnerable to targeted disruption of CDK7 kinase activity and RNAPII processivity, underscoring their reliance on hyperactivated transcriptional amplification^[20]^. THZ1, the first identified selective and irreversible CDK7 inhibitor, has demonstrated efficacy in preclinical models of malignancies driven by TF dysregulation, including GBM^[21]^. Targeting CDK7 with THZ1 disrupts global transcriptional networks in GBM cells, preferentially abrogating SE-driven oncogenic circuits and dismantling core resistance mechanisms^[22]^. However, THZ1’s short half-life (45 min) limits its clinical application^[23]^. In contrast, THZ2, a structural derivative of THZ1, exhibits a 5-fold longer plasma half-life. THZ2 induces complete ablation of Ser-2/5/7 phosphorylation on the RNAPII carboxyl-terminal domain through irreversible covalent engagement with CDK7, resulting in systemic transcriptional repression^[24]^. This inhibition of CDK7-mediated phosphorylation also impairs the phosphorylation of downstream CDKs, ultimately leading to cell cycle arrest and apoptosis^[25,26]^. THZ2 has demonstrated significant antitumor efficacy in multiple malignancies, including breast cancer^[25]^ and osteosarcoma^[27]^. However, its anticancer potential in GBM remains largely unexplored. BRD4, a key member of the bromodomain and extra-terminal (BET) family of proteins, is also considered a promising therapeutic target for SE modulation. Both CDK7 and BRD4 inhibition preferentially disrupt transcription at SE-associated genes. JQ1, a small-molecule inhibitor targeting BRD4, has shown efficacy in various tumors and demonstrates synergistic effects when combined with other inhibitors^[28]^.

In this study, we explored the anti-GBM effects of THZ2 through CDK7inhibition, using various *in vitro* and *in vivo* assays. Our findings reveal that THZ2 significantly suppresses the proliferation, migration, and invasion of GBM cells, induces cell cycle arrest, and promotes apoptosis. Moreover, THZ2 exhibits a synergistic antitumor effect when combined with TMZ and is capable of reversing chemoresistance in GBM cells. We also demonstrated that JQ1, an inhibitor of BRD4, exerts synergistic cytotoxicity with TMZ in GBM cells. Finally, we identified SE-related genes potentially involved in GBM chemoresistance through literature analysis, further elucidating the potential anti-GBM mechanisms of THZ2.

## METHODS

### Cell culture

Human GBM cell lines (A172, U118MG, U87MG, and U251), obtained from the Cell Bank of the Chinese Academy of Sciences, were cultured in DMEM/high-glucose medium containing 10% fetal bovine serum (Cellmax, China), 100 U/mL penicillin, and 100 ng/mL streptomycin (Beyotime, China). Cells were maintained at 37 °C in a humidified atmosphere containing 5% CO_2_.

### Compounds and reagents

THZ2 (BCP24675) and JQ1 (BCP20870) were purchased from BioChemPartner (Shanghai, China). TMZ (HY-17364), a chemotherapeutic agent used for GBM, was purchased from MedChemExpress (MCE, USA).

### Establishment of TMZ-resistant cell lines

Log-phase GBM cells were seeded in 96-well plates (5 × 10^3^ cells/well), and the IC_50_ of TMZ for U87MG cells was determined to be 1.21 mM. Cells were initially exposed to TMZ at 1/100 of the IC_50_ (0.0121 mM), followed by stepwise increases (0.02, 0.04, 0.08, 0.16, 0.32, 0.64, 1.0 mM) upon cellular adaptation. Each concentration was maintained for 14 days before escalation.

### Cell viability assay

Cell viability was assessed using a CCK-8 assay kit (APExBIO, China). GBM cells were seeded in 96-well plates at 5 × 10^3^ cells/well for standard assays or 2 × 10^3^ cells/well for time-course studies and treated with gradient concentrations of THZ2 or TMZ. At specified time points, 100 μL of 10% CCK-8 solution was added per well and incubated for 1 h at 37 °C. Absorbance was measured at 450 nm using a microplate reader, and results were averaged from triplicate wells.

### Colony formation assay

GBM cells were seeded in triplicate at 700 cells/well in 6-well plates containing 2 mL DMEM with 10% FBS. Once cells formed visible colonies (two to three per individual cell), they were treated with dimethylsulfoxide (DMSO, Beyotime, China) or various concentrations of THZ2, with media changed every three days. After 10 days, cells were fixed with methanol and stained with 0.1% (w/v) crystal violet solution (Beyotime, China). Colonies containing more than 50 cells were counted and photographed. Data are presented as mean ± standard deviation (SD) from three independent experiments performed in triplicate.

### *In vitro* migration and invasion assays

Cell migration and invasion were assessed using Transwell chambers with 8-μm pores (6.5 mm diameter; JET BIOFIL, China). Chambers were left uncoated for migration assays or precoated with Matrigel (1:10 dilution in DMEM; BD Biosciences, USA) for invasion assays. After 24 h of serum starvation, 200 μL of serum-free DMEM containing 5 × 10^4^ cells/mL was added to the upper chambers, and 650 μL of DMEM with 20% FBS was added to the lower chambers. Cells were treated with gradient concentrations of THZ2 or 0.5 mM TMZ. After 48 h at 37 °C, migrated/invaded cells were fixed with 4% paraformaldehyde for 30 min, stained with 0.1% crystal violet for 30 min, and counted in five random fields using an inverted microscope (Olympus Corporation, Japan). Data (mean ± SD) were analyzed using ImageJ and GraphPad Prism.

### Cell cycle assays

Cell cycle distribution was analyzed using a commercial kit (Beyotime, China). Cells (5 × 10^4^) were washed with PBS, fixed in ice-cold 70% ethanol at 4 °C for 4 h, and centrifuged at 1,000 × *g* for 5 min. After PBS washing, cell pellets were resuspended in 0.5 mL of PI/RNase A staining buffer, incubated at 37 °C for 30 min in the dark, and analyzed by flow cytometry (excitation at 488 nm). Data were processed with FlowJo 10.6.2 software.

### Apoptosis assays

Apoptosis was assessed using an Annexin V-FITC/propidium iodide (PI) dual-staining assay kit (Beyotime, China). Cells (1 × 10^5^) were collected, resuspended in binding buffer, and stained with Annexin V-FITC and PI. Samples were analyzed by flow cytometry, and data were processed using FlowJo 10.6.2.

### Drug synergy studies

Synergy between TMZ and THZ2/JQ1 was evaluated using a 3 × 3 concentration matrix with 48-hour incubation. Cell viability was assessed via CCK-8 assay in triplicate. SynergyFinder software (https://synergy-finder.fimmm.fi) was used to generate response surface heatmaps, with synergistic effects quantified using ZIP synergy scores: ZIP > 0 indicates synergy; ZIP = 0 indicates an additive effect; ZIP < 0 indicates antagonism; ZIP > 10 indicates strong synergy. The combination index (CI) was also calculated using CompuSyn software (http://www.combosyn.com/), with CI ≤ 0.9 considered synergistic, 0.9 ≤ CI ≤ 1.1 considered additive, and CI ≥ 1.1 considered antagonistic^[29]^.

### RNA extraction and quantitative real-time PCR

Total RNA was extracted using Trizol reagent (Beyotime, China). cDNA was synthesized from 1 μg RNA using oligo-dT_18_ primers and Multiscribe Reverse Transcriptase. RT-qPCR was performed on a QuantStudio 6 Flex system using SYBR Green Master Mix (Yeasen, China). Relative gene expression was calculated using the ΔΔCt method, normalized to GAPDH, and performed in triplicate. The qPCR primer sequences were: SOX9: AGCGAACGCACATCAAGAC, CTGTAGGCGATCTGTTGGGG, GAPDH: TCCAAAATCAAGTGGGGCGA, TGGTTCACACCCATGACGAA.

### Western blot analysis

Cells were washed with PBS and lysed on ice for 5 min in RIPA buffer supplemented with protease/phosphatase inhibitors (BOSTER, China). Lysates were collected, further incubated at 4 °C for 30 min, and centrifuged at 12,000 × *g* for 15 min. Protein concentrations were determined using a Bradford assay. Equal protein aliquots were denatured by boiling for 10 min, separated via SDS-PAGE, and transferred to PVDF membranes (Millipore, USA). Membranes were blocked with 5% non-fat milk in TBST for 1 h at room temperature, incubated overnight at 4 °C with primary anti-SOX9 antibody (1:1,000, AF2329, Beyotime, China), followed by TBST washes and 1 h incubation with HRP-conjugated secondary antibody at room temperature. Protein bands were visualized using a ChemiDoc imaging system (Bio-Rad) and quantified using ImageJ from three independent experiments.

### CUT&RUN

The CUT&RUN assay was conducted using the pG-MNase CUT&RUN profiling kit (Vazyme, China) following the manufacturer’s instructions. Briefly, 1 × 10^5^ cells were harvested, bound to activated ConA Beads Pro, and incubated overnight at 4 °C with either an anti-H3K27ac antibody (39134, Proteintech, China) or rabbit IgG (Proteintech, China). Samples were treated with pG-MNase at 4 °C for 1 h, followed by calcium chloride addition and further incubation for 60 min at 4 °C to induce chromatin digestion. Released DNA fragments were then purified for downstream analysis.

The primer sequences for CUT&RUN-PCR/qPCR were: CDK7-F: TAGCACTTATCACCTATGTATCA, CDK7-R: GCACCATTGTAGAATCTGAG. BRD4-F: GAGCTACCCACAGAAGAAACC, BRD4-R: GAGTCGATGCTTGAGTTGTGTT. SOX9-F: TGTGTCTCCGCTCCCGG, SOX9-R: ATTTCTGCAGGGGCCTCCTG.

### Tumor xenograft models

All animal experiments were approved by the Ethics Committee of the Second Xiangya Hospital, Central South University (Approval NO. 2020031). Subcutaneous U87 xenografts were established in the limbs of nude mice. Once tumors reached ~100 mm^3^, mice were randomized into four groups: Control, TMZ (50 mg/kg i.p. every other day), THZ2 (10 mg/kg i.p. daily), and TMZ + THZ2 combination. Body weight and tumor volume were recorded at least 3 times weekly. After 14 days of treatment, mice were euthanized, and tumors and major organs were collected for formalin fixation and hematoxylin-eosin (H&E) staining.

### Statistical analysis

Statistical analyses were performed using GraphPad Prism version 9.3.1. Data are presented as mean ± SD from at least three independent experiments. Statistical significance was assessed using Student’s *t*-test or one-way/two-way analysis of variance (ANOVA), as appropriate. Significant levels are indicated as follows: ^*^P < 0.05; ^**^P < 0.01; ^***^P < 0.001. Figures were prepared using Photoshop.

## RESULTS

### THZ2 suppresses GBM cell proliferation, migration, and invasion

**Figure 1:**
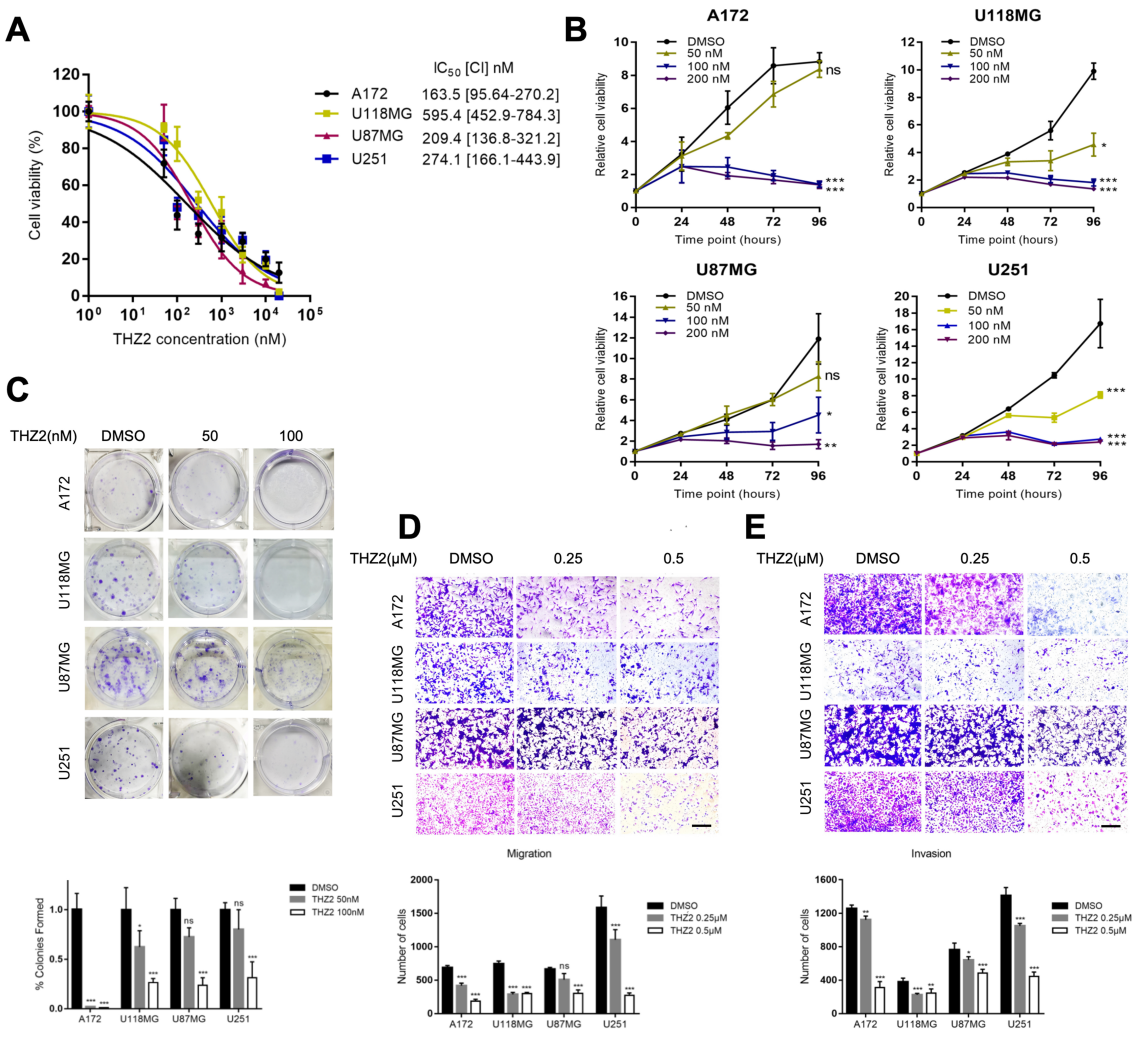
THZ2 suppresses GBM cell proliferation, migration, and invasion. (A) IC_50_ values of THZ2 in four GBM cell lines; (B and C) Effects of different concentrations of THZ2 on proliferation (B) and colony formation (C) in four GBM cell lines; (D and E) Effects of THZ2 (0.25-0.5 μM for 48 h) on migration (D) and invasion (E) in four GBM cell lines. Scale bars: 50 μM. Data are presented as mean ± SD from three independent experiments (*n* = 3). ns: Not significant, ^*^P < 0.05, ^**^P < 0.01, ^***^P < 0.001. Statistical significance was assessed using one-way ANOVA. GBM: Glioblastoma; SD: standard deviation; ANOVA: analysis of variance.

Dose-response experiments were conducted to evaluate the effects of THZ2 on GBM cells. These experiments revealed that all four GBM cell lines tested were highly sensitive to THZ2, with IC_50_ values ranging from 57.6 to 719.4 nM [Figure 1A]. THZ2 inhibited the proliferation of A172, U118MG, U87MG, and U251 cells in a time-dependent manner, with notable effects observed at concentrations as low as 50, 100, or 200 nM [Figure 1B]. Colony formation assays further confirmed the anti-proliferative activity of THZ2, as it markedly reduced colony formation in these cells [Figure 1C]. Consistently, THZ2 treatment significantly suppressed both the migration [Figure 1D] and invasion [Figure 1E] abilities of the four GBM cell lines. Collectively, these results indicate that THZ2 effectively inhibits GBM cell proliferation, migration, and invasion.

### THZ2 induces cell cycle arrest and apoptosis in GBM cells

**Figure 2:**
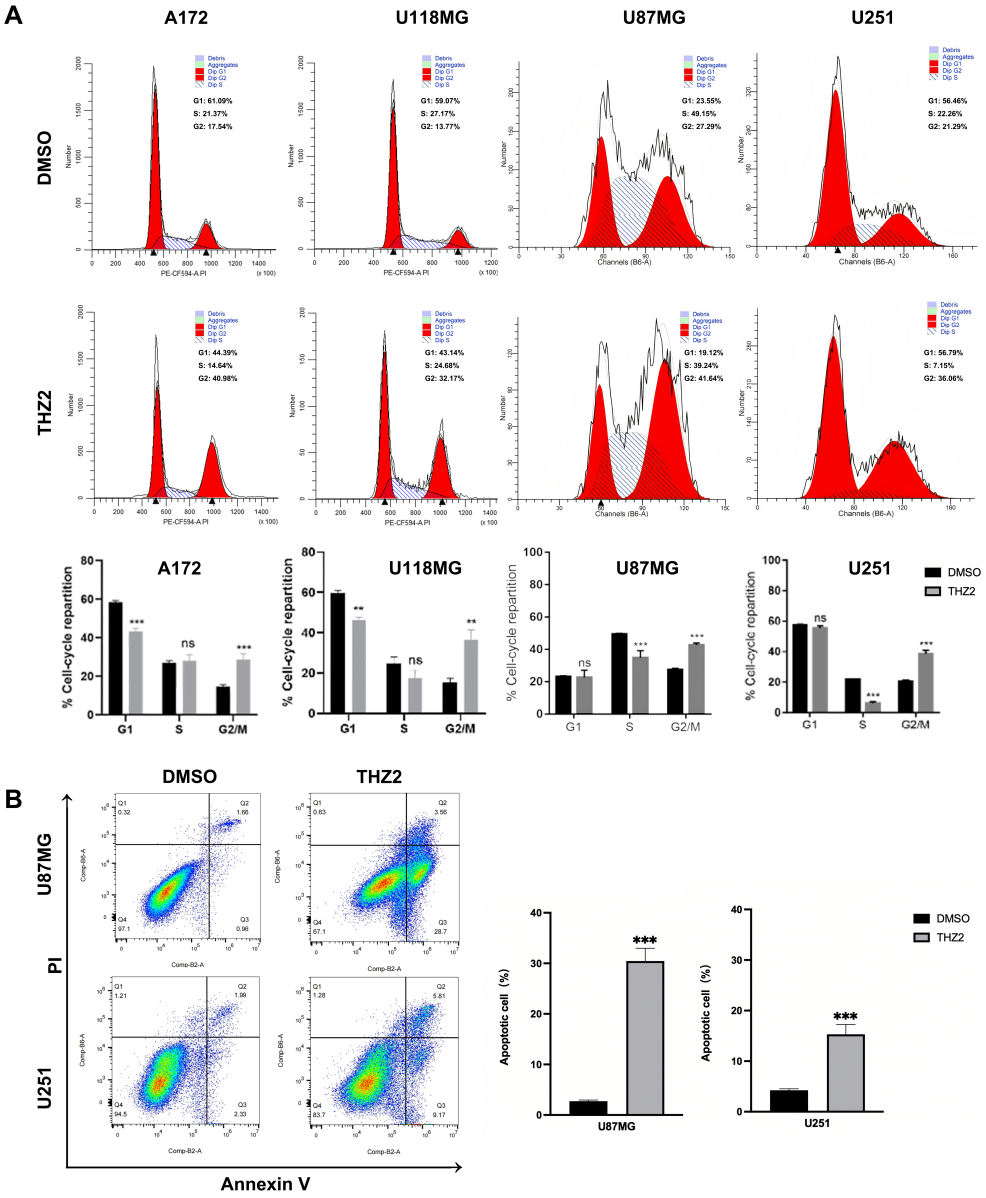
THZ2 induces dual cytotoxic effects in GBM cells: cell cycle arrest and apoptosis. (A and B) Effects of 0.25 μM THZ2 for 48 h on cell cycle distribution (A) and apoptosis (B) in A172, U118MG, U87MG, and U251 cells. Data are presented as mean ± SD from three independent experiments (*n* = 3). ns: Not significant; ^**^P < 0.01; ^***^P < 0.001. Statistical significance was assessed using Student’s *t*-test. GBM: Glioblastoma; SD: standard deviation.

To investigate whether THZ2-induced growth inhibition is mediated by cell cycle arrest, the four GBM cell lines (A172, U118MG, U87MG, and U251) were treated with 0.25 μM THZ2 for 24 h. Flow cytometry analysis using PI staining revealed consistent accumulation of cells in the G2-M phase and a reduction in the S phase across all cell lines. Specifically, the percentages of cells in G2-M increased by 23.44%, 18.40%, 14.35%, and 14.77% for A172, U118MG, U87MG, and U251, respectively, while the percentages in S phase decreased by 6.73%, 2.49%, 9.91%, and 15.11%, respectively [Figure 2A].

To further determine whether THZ2 induces apoptosis, U87MG and U251 cells were treated with 0.25 μM THZ2 for 48 h, followed by flow cytometry analysis using Annexin V/PI staining. As shown in Figure 2B, the proportion of apoptotic cells increased by 34.17% in U87MG cells and by 13.23% in U251 cells [Figure 2B]. These findings demonstrate that THZ2 not only induces cell cycle arrest but also promotes apoptosis in GBM cells.

### Co-treatment with THZ2/JQ1 and TMZ synergistically inhibits GBM cell viability

**Figure 3:**
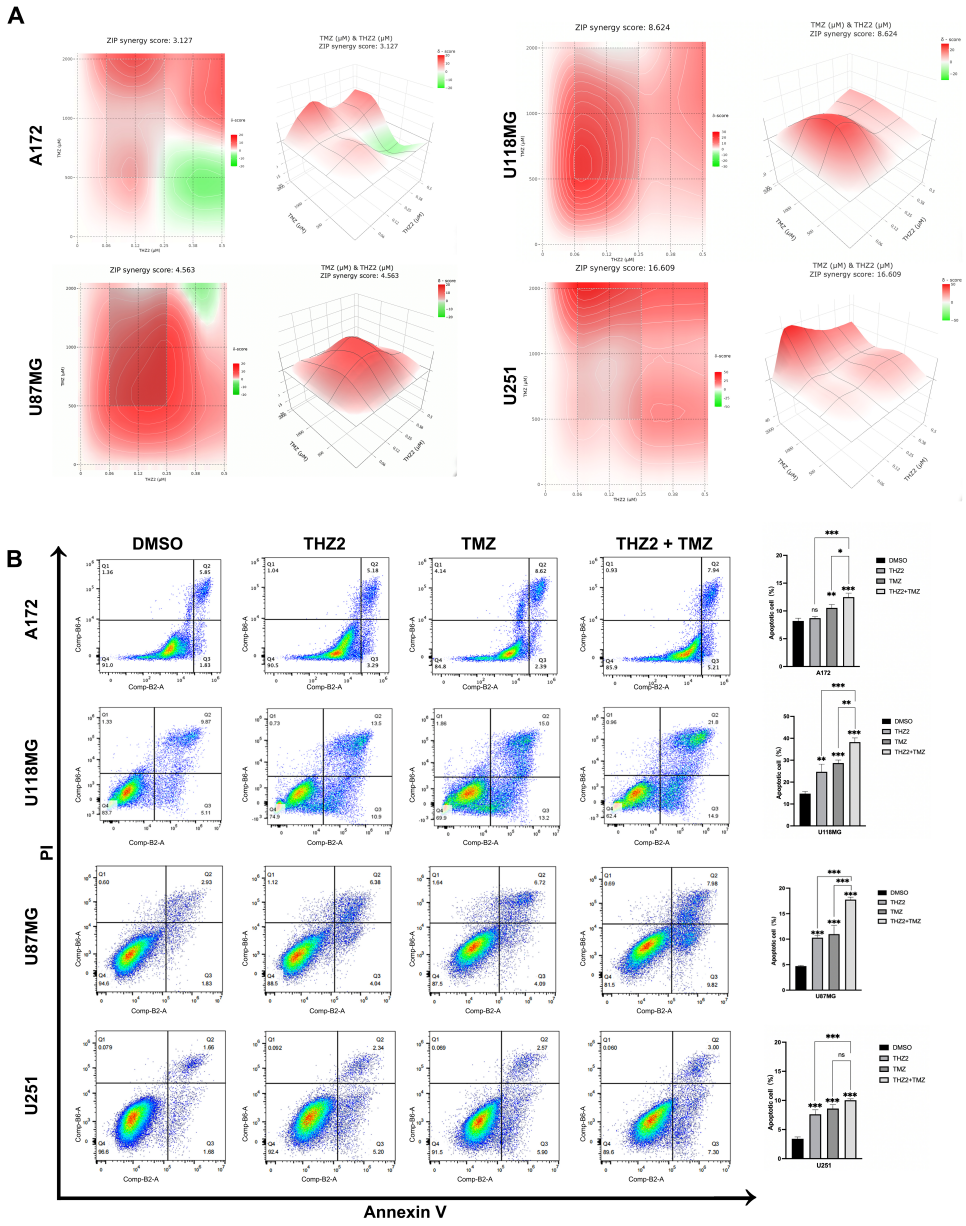
THZ2 and TMZ exhibit synergistic anti-GBM effects. (A and B) Effects of co-treatment with 0.1 μM THZ2 and 0.5 mM TMZ for 48 h on apoptosis in A172, U118MG, U87MG, and U251 cells. In panel (A), the tested THZ2 concentrations were 0.0625, 0.125, 0.25, 0.375, and 0.5 μM, respectively, and the TMZ concentrations were 0.5, 1, and 2 mM. Data are presented as mean ± SD from three independent experiments (*n* = 3). ns: No significant; ^*^P < 0.05; ^**^P < 0.01; ^***^P < 0.001. Statistical significance was determined using one-way ANOVA. TMZ: Temozolomide; GBM: glioblastoma; SD: standard deviation; ANOVA: analysis of variance.

Drug synergy occurs when combination therapy enhances efficacy at lower doses, thereby reducing adverse effects. TMZ is the most commonly used chemotherapeutic agent for treating gliomas. To investigate whether small-molecule inhibitors targeting SEs can increase the sensitivity of GBM cells to TMZ, we examined the synergistic effects of THZ2 and TMZ on the viability of A172, U118MG, U87MG, and U251 cell lines. Cells were treated with varying concentrations of THZ2 and/or TMZ for 48 h, and cell viability was assessed using the CCK-8 assay. We calculated CIs and found that THZ2 exhibited varying degrees of synergy with TMZ across all four cell lines, with ZIP scores of 3.127, 8.624, 4.563, and 16.609, respectively. Notably, in U251 cells, all tested concentration combinations demonstrated synergistic effects [Figure 3A]. These findings suggest that THZ2 enhances the sensitivity of GBM cells to TMZ. We further evaluated the effect of THZ2 and TMZ co-treatment on apoptosis using the Annexin V/PI apoptosis detection kit. Flow cytometry analysis revealed that the combination significantly increased apoptosis in A172, U118MG, U87MG, and U251 cells [Figure 3B].

To further assess the therapeutic potential of small-molecule epigenetic inhibitors targeting SEs in GBM, we tested JQ1, a BRD4 inhibitor, for its effect on GBM cell viability, both alone and in combination with TMZ. Similar to THZ2, JQ1 also inhibited the viability of all four GBM cell lines [Supplementary Figure 1]. We then evaluated the synergistic effects of JQ1 and TMZ. The combination significantly reduced cell viability in U87MG and U251 cells [Table 1]. Collectively, these results indicate that combining THZ2 or JQ1 with TMZ significantly enhances anti-GBM effects, demonstrating synergistic interactions between these compounds [Table 2].

**Table 1:** CI of JQ1 and TMZ in U87MG and U251 cells

**Concentration**		**U87MG**		**U251**	
**TMZ (mM)**	**JQ1 (μM)**	**Effect**	**CI**	**Effect**	**CI**
0	10	0.06	/	0.25	/
0	20	0.03	/	0.32	/
0	40	0.36	/	0.58	/
0.5	0	0.23	/	0.30	/
0.5	10	0.27	1.11	0.46	0.89
0.5	20	0.30	1.23	0.57	0.80
0.5	40	0.69	0.51	0.66	0.86
1	0	0.42	/	0.51	/
1	10	0.50	0.88	0.68	0.55
1	20	0.53	0.91	0.72	0.57
1	40	0.83	0.38	0.85	0.37
2	0	0.61	/	0.61	/
2	10	0.68	0.86	0.81	0.46
2	20	0.77	0.65	0.82	0.48
2	40	0.94	0.24	0.85	0.52

CI: Combination index; TMZ: temozolomide.

**Table 2:** CI of THZ2 and TMZ in U87MG and U251 cells

**Concentration**		**U87MG**		**U251**	
**TMZ (mM)**	**THZ2 (μM)**	**Effect**	**CI**	**Effect**	**CI**
0	0.125	0.49	/	0.40	/
0	0.25	0.52	/	0.45	/
0	0.5	0.61	/	0.48	/
0.5	0	0.18	/	0.25	/
0.5	0.125	0.57	0.64	0.45	0.85
0.5	0.25	0.64	0.56	0.50	0.70
0.5	0.5	0.62	1.02	0.56	0.52
1	0	0.50	/	0.44	/
1	0.125	0.66	0.53	0.55	0.60
1	0.25	0.68	0.57	0.59	0.51
1	0.5	0.67	0.85	0.63	0.44
2	0	0.56	/	0.54	/
2	0.125	0.70	0.78	0.63	0.74
2	0.25	0.64	1.23	0.66	0.65
2	0.5	0.64	1.79	0.68	0.60

CI: Combination index; TMZ: temozolomide.

### THZ2/JQ1 exhibits therapeutic potential in TMZ-resistant GBM cells

**Figure 4:**
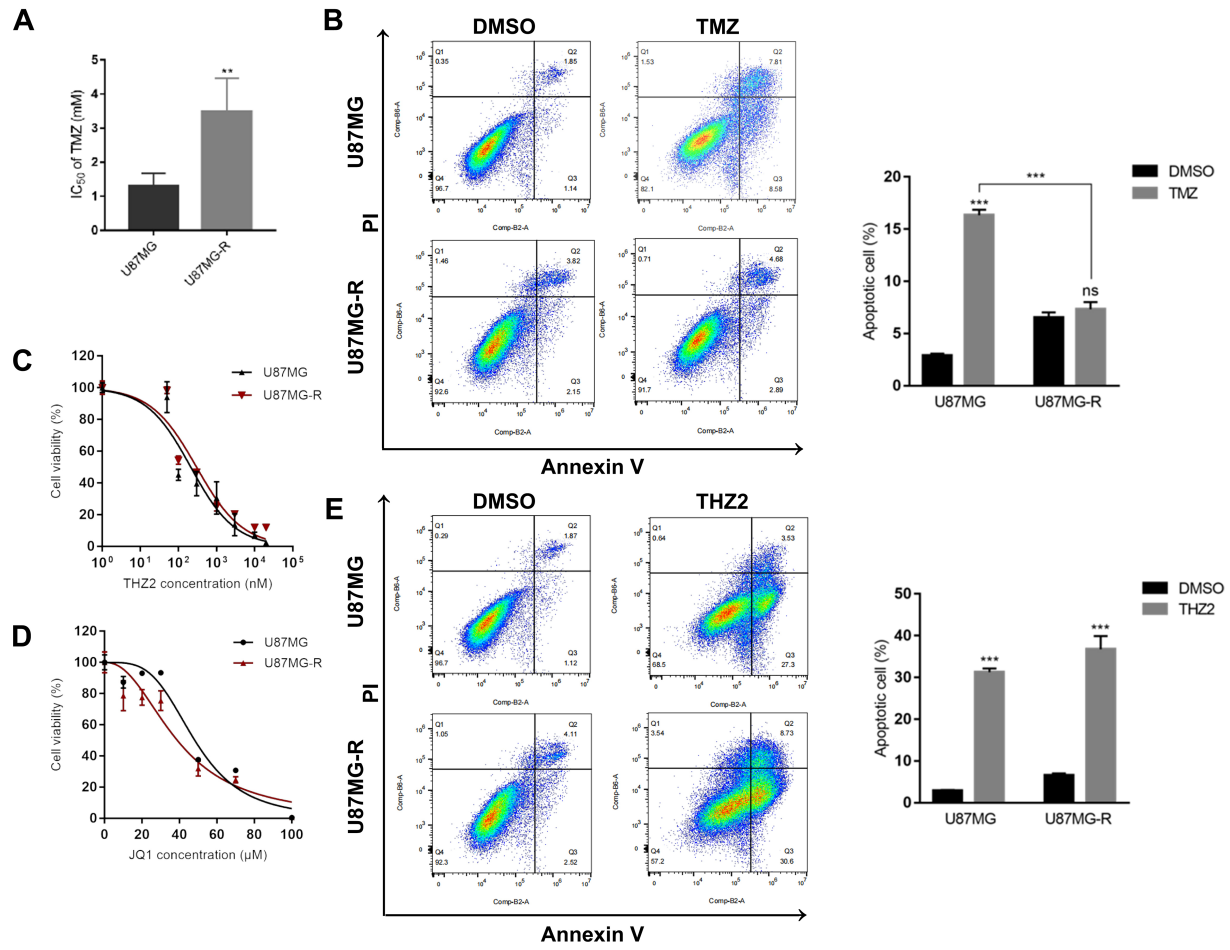
THZ2/JQ1 exhibits therapeutic potential in TMZ-resistant GBM cells. (A) IC_50_ values of TMZ in U87MG and U87MG-R cells; (B) Effects of 1 mM TMZ treatment for 48 h on apoptosis in U87MG and U87MG-R cells; (C and D) Effects of different concentrations of THZ2 (C) and JQ1 (D) on U87MG-R cell viability; (E) Effects of 0.25 μM THZ2 treatment for 48 h on apoptosis in U87MG and U87MG-R cells. Data are presented as mean ± SD from three independent experiments (*n* = 3). ns: No significant; ^**^P < 0.01; ^***^P < 0.001. Statistical significance was assessed using Student’s *t*-test or two-way ANOVA. TMZ: Temozolomide; GBM: glioblastoma; SD: standard deviation; ANOVA: analysis of variance.

As treatment with TMZ often leads to the development of resistance, we further investigated the mechanisms behind TMZ resistance in GBM cells. We hypothesized that SEs contribute to TMZ resistance in GBM. To test this, we established a TMZ-resistant U87MG cell line, designated U87MG-R, by continuously exposing the cells to TMZ for 6 months. The resulting resistance index was approximately 3.5 [Figure 4A]. TMZ exerts antitumor effects by blocking the initiation of DNA replication and inducing apoptosis. We confirmed the chemoresistant phenotype of U87MG-R cells using an apoptosis assay. The results showed that TMZ induced a significantly higher rate of apoptosis in parental U87MG cells compared to U87MG-R cells [Figure 4B].

As shown in Figure 4C and D, U87MG-R cells remained sensitive to both THZ2 and JQ1. Notably, treatment with THZ2 significantly increased apoptosis in U87MG-R cells [Figure 4E]. To assess whether THZ2 could synergistically enhance TMZ sensitivity in resistant cells, we treated U87MG-R cells with various concentrations of THZ2 and TMZ for 48h and performed a CCK-8 assay. The results demonstrated that THZ2 sensitized U87MG-R cells to TMZ, with the combination treatment synergistically inhibiting cell proliferation [Table 3]. Moreover, this synergistic effect was more pronounced in resistant cells than in sensitive cells. These findings suggest that TMZ resistance in GBM cells is highly dependent on SE activity.

**Table 3:** CI of THZ2 and TMZ in U87MG-R cells

**Concentration**		**U87MG-R**	
**TMZ (mM)**	**THZ2 (μM)**	**Effect**	**CI**
0	0.125	0.55	/
0	0.25	0.55	/
0	0.5	0.63	/
0.5	0	0.09	/
0.5	0.125	0.69	0.25
0.5	0.25	0.67	0.38
0.5	0.5	0.63	1.01
1	0	0.38	/
1	0.125	0.74	0.34
1	0.25	0.68	0.53
1	0.5	0.71	0.52
2	0	0.51	/
2	0.125	0.73	0.68
2	0.25	0.71	0.77
2	0.5	0.71	0.86

CI: Combination index; TMZ: temozolomide.

### THZ2 significantly inhibits subcutaneous GBM tumor growth

**Figure 5:**
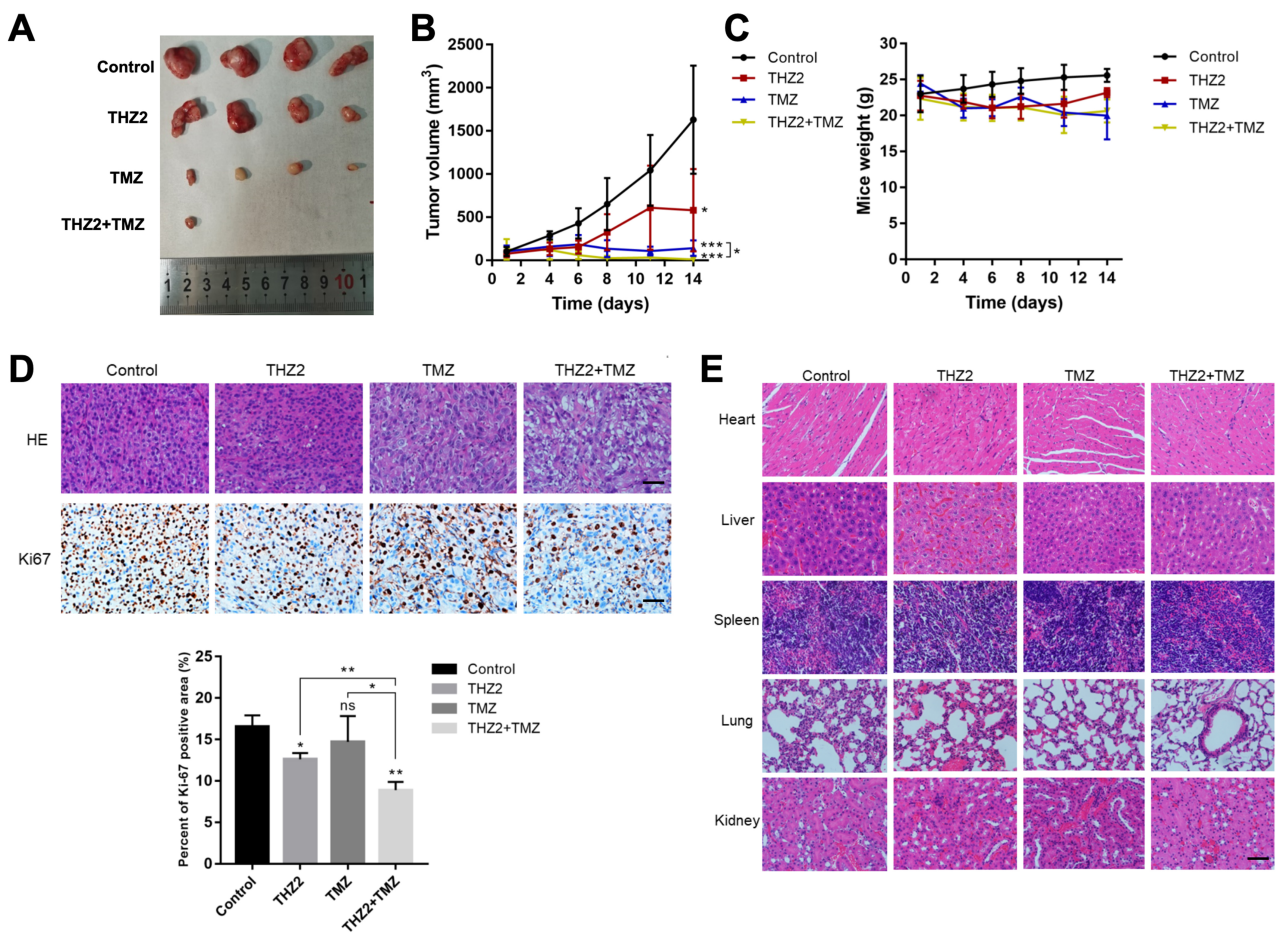
THZ2 significantly inhibits subcutaneous GBM tumor growth. (A and B) THZ2 in combination with TMZ inhibits the growth of subcutaneous GBM tumors in nude mice (*n* = 4); (C) Effects of THZ2 and TMZ on body weight in nude mice; (D) Effects of THZ2, TMZ, or their combination on Ki67 expression in subcutaneous GBM tissue; (E) Histological examination of major organs (heart, liver, spleen, lung, and kidney) in mice treated with THZ2, TMZ, or their combination. Scale bars: 20 μM. Data are presented as mean ± SD from three independent experiments (*n* = 3). ns: No significant; ^*^P < 0.05; ^**^P < 0.01; ^***^P < 0.001. Statistical significance was assessed using one-way ANOVA. GBM: Glioblastoma; TMZ: temozolomide; SD: standard deviation; ANOVA: analysis of variance.

Subcutaneous U87MG xenografts in nude mice were established to evaluate the *in vivo* antitumor efficacy of THZ2 and TMZ combination therapy. Both THZ2 and TMZ alone significantly inhibited tumor growth compared to the control group. Although TMZ at sufficient doses markedly suppressed tumor growth, the combination of THZ2 and TMZ resulted in even greater inhibition, indicating a synergistic effect [Figure 5A and B]. No significant weight loss was observed in mice receiving the combination treatment, suggesting that the co-administration of THZ2 and TMZ does not cause obvious systemic toxicity [Figure 5C]. Immunohistochemical analysis revealed that Ki67 expression - a marker of cell proliferation - was lower in the combination group compared to both the control and single-agent groups [Figure 5D]. Additionally, hematoxylin and eosin (HE) staining of major organs (heart, liver, spleen, lung, and kidney) showed no significant pathological changes in mice treated with THZ2 or the combination therapy, further supporting the safety of THZ2 at the tested dose [Figure 5E]. Collectively, these findings indicate that THZ2 synergistically enhances the inhibitory effect of TMZ on GBM tumor growth *in vivo* without inducing notable side effects.

### THZ2 inhibits the expression of SOX9 in GBM cells

**Figure 6:**
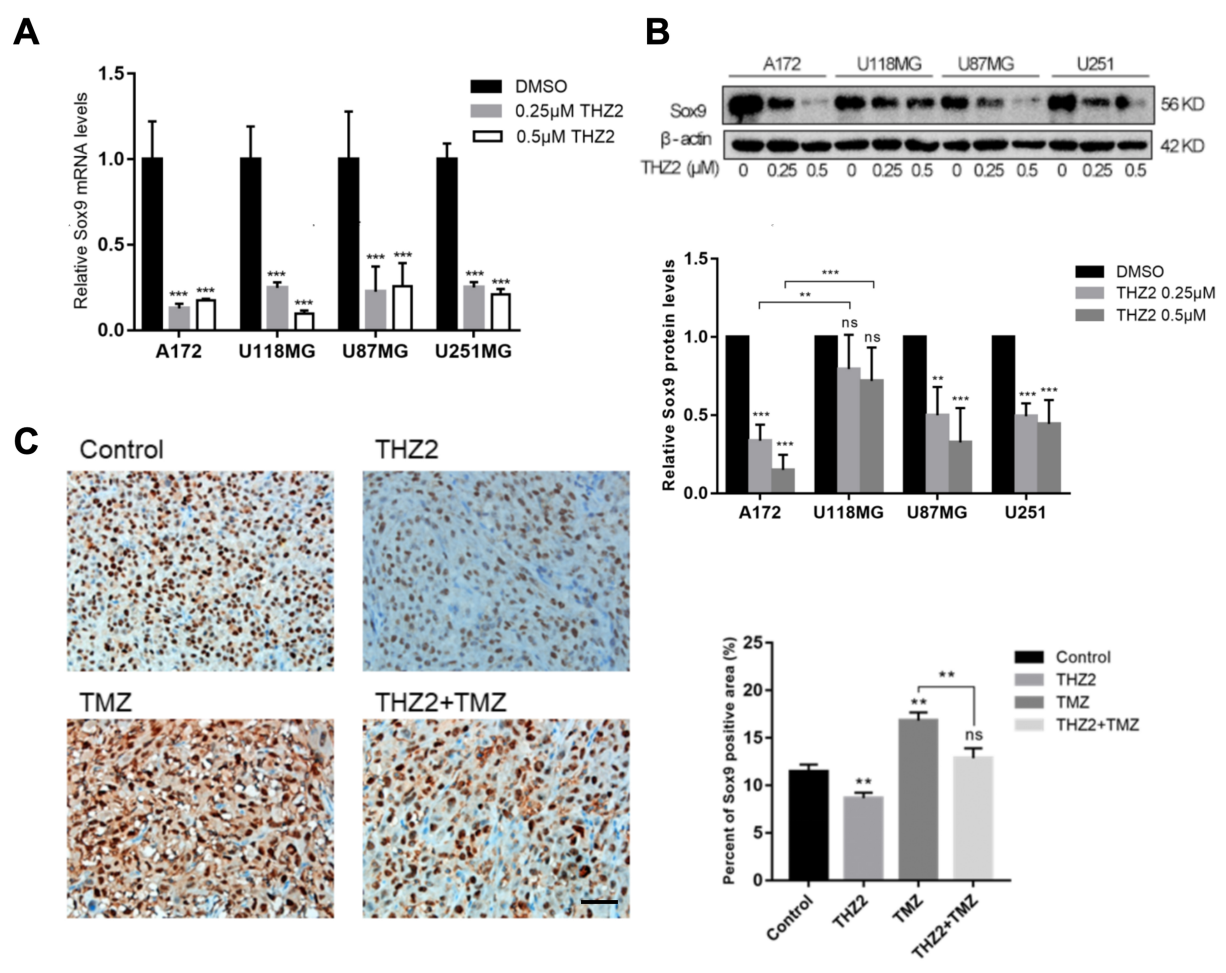
THZ2 inhibits SOX9 expression in GBM cells. (A) Protein levels of SOX9 following 48-hour treatment with 0.25-0.5 μM THZ2 in four GBM cells (*n* = 3); (B) mRNA levels of SOX9 following the same treatment (*n* = 3); (C) Effects of THZ2 and TMZ, alone or in combination, on SOX9 expression in subcutaneous GBM tissue. Scale bars: 20 μM. Data are presented as mean ± SD from three independent experiments (*n* = 3). ns: No significant; ^**^P < 0.01; ^***^P < 0.001. Statistical significance was assessed using one-way ANOVA. GBM: Glioblastoma; TMZ: temozolomide; SD: standard deviation; ANOVA: analysis of variance.

SOX9 is a key SE-regulated TF involved in the regulation and maintenance of various stemness-related genes, including MAFB^[30]^, NUMB^[31]^, NOTCH2^[32]^, FOXC1^[33]^, and ALDH1A1^[34]^. We further investigated whether THZ2 enhances the sensitivity of GBM cells to TMZ by modulating SOX9 expression. To assess whether SEs drive SOX9 expression in GBM, we treated GBM cell lines with THZ2 and evaluated its impact on SOX9 expression. We observed that SOX9 mRNA levels were significantly reduced following THZ2 treatment. Although SOX9 protein levels in U118MG cells also declined in a dose-dependent manner, the reduction was less pronounced compared to other GBM cell lines [Figure 6A and B]. Additionally, immunohistochemical analysis of subcutaneous tumor tissues revealed that THZ2 treatment decreased SOX9 expression, whereas TMZ treatment increased it. Interestingly, combined treatment showed no significant difference compared to the control group [Figure 6C].

**Figure 7:**
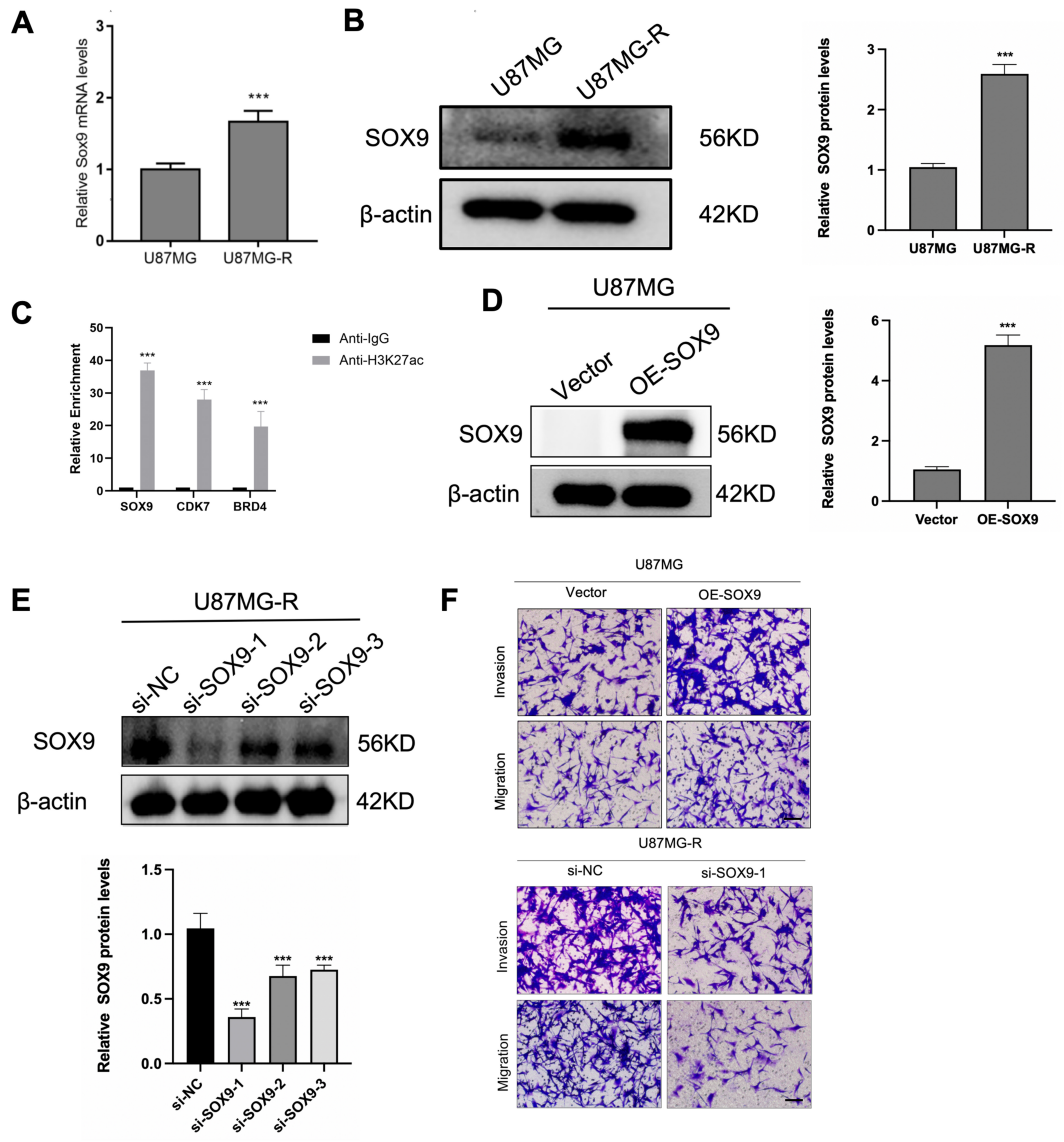
SOX9 regulates drug sensitivity through effects on GBM cell migration and invasion. (A and B) mRNA and protein expression of SOX9 in U87MG and U87MG-R cells (*n* = 3); (C) CUT&RUN analysis showing genes enriched with H3K27ac in U87MG-R cells; (D) Protein levels of overexpressed SOX9 in U87MG cells; (E) Protein levels of silenced SOX9 in U87MG-R cells; (F) Transwell assays to detect the migration and invasion in OE-SOX9 and si-SOX9 cells (*n* = 3). Scale bars: 20 μM. Data are presented as mean ± SD from three independent experiments (*n* = 3). ^***^P < 0.001. Statistical significance was assessed using Student’s *t*-test or one-way ANOVA. GBM: Glioblastoma; SD: standard deviation; ANOVA: analysis of variance.

Given the established link between tumor stemness and drug resistance, we analyzed SOX9 expression in paired TMZ-sensitive and TMZ-resistant GBM cell pairs. Both mRNA and protein levels of SOX9 were markedly elevated in TMZ-resistant U87MG-R cells compared to parental U87MG cells [Figure 7A and B]. To explore this further, we performed CUT&RUN assays in TMZ-resistant cells with high SOX9 expression. Using an anti-H3K27ac antibody for target DNA enrichment, we assessed the chromatin binding of SOX9, CDK7, and BRD7. The results demonstrated significant enrichment of chromatin DNA associated with these factors compared to the IgG control [Figure 7C], supporting the role of SOX9 as a SE-regulated gene involved in TMZ resistance. Next, we overexpressed SOX9 in U87MG cells and knocked down SOX9 in U87MG-R cells [Figure 7D and E]. Transwell migration and invasion assays were conducted to assess functional changes. Under identical TMZ treatment conditions, the SOX9 overexpression (OE-SOX9) group showed increased cell migration and invasion, while the SOX9 silence (si-SOX9) group exhibited significantly reduced migratory and invasive capacities compared to their respective controls [Figure 7F]. These findings confirm that SOX9 contributes to both drug sensitivity and the malignant phenotype of GBM cells by promoting migration and invasion, reinforcing its functional role as a resistance-related gene.

## DISCUSSION

Despite advances in multimodal treatments, including surgery, radiation, and chemotherapy, GBM remains characterized by aggressive behavior and resistance to therapy, leading to frequent recurrence and limited overall survival^[5]^. Therefore, elucidating the molecular mechanisms underlying GBM progression and chemoresistance, and identifying more effective treatment strategies are of paramount importance. Our study demonstrates that THZ2 inhibits the proliferation, migration, and invasion of GBM cells, induces cell cycle arrest, and promotes apoptosis.

Radiotherapy combined with TMZ is the established first-line adjuvant therapy for GBM; however, resistance to TMZ frequently develops. This highlights the urgent need for novel approaches to overcome chemoresistance. SEs, which are known to influence GBM progression and drug resistance, primarily involve CDK7 and BRD4 as key components. CDK7 functions through two main pathways: as part of the CDK-activating kinase (CAK) complex with CyclinH and MATI, it phosphorylates CDKs 1, 2, 4, and 6 to regulate the cell cycle; and as a component of the basal transcription factor TFIIH, it phosphorylates serine residues at positions 5 and 7 of the carboxy-terminal domain of RNAPII, facilitating transcription initiation^[35,36]^. THZ2, a selective covalent inhibitor of CDK7, has shown significant synergy with TMZ *in vitro*. The notable anti-GBM efficacy of the THZ2/TMZ combination supports its potential for clinical translation and drug repurposing. Moreover, TMZ-resistant cells retained sensitivity to THZ2, suggesting promising translational potential for targeting chemoresistance in GBM using chromatin-targeting small molecules. BRD4, a member of the BET protein family, is another SE-associated therapeutic target. Inhibitors of CDK7 and BRD4 can disrupt transcription of SE-associated oncogenes. JQ1, a small-molecule BRD4 inhibitor, has demonstrated efficacy across various tumor types and synergizes with other targeted therapies. Consistent with existing literature^[37]^, our study found that JQ1 combined with TMZ exerts synergistic cytotoxicity in GBM cells.

SOX9, an oncogene, is overexpressed in various malignancies, including lung^[38]^, prostate^[39]^, and colorectal cancers^[40]^, and is also implicated in drug resistance across tumor types^[41]^. Previous studies have shown elevated SOX9 expression in GBM cell lines compared to normal astrocytes^[42]^, with high SOX9 levels correlating with poor prognosis^[43]^. Importantly, TMZ has been reported to upregulate SOX2 and SOX9 in U87MG cells, with these elevated levels contributing to TMZ resistance. Rapamycin can suppress SOX protein expression, and its combination with TMZ inhibits the growth of cells with high SOX2/SOX9 expression^[44]^. Direct knockdown of SOX9 in U87MG and U251 cells has been shown to increase TMZ sensitivity, potentially via the miR-138-5p/SOX9 axis^[45]^. Our results indicate that SOX9 mRNA levels increase in TMZ-resistant GBM cells, supporting its involvement in chemoresistance, consistent with previous findings^[44]^. Additionally, we observed that THZ2 reduces SOX9 protein levels in GBM cells, suggesting that CDK7 inhibition may enhance TMZ sensitivity by suppressing SOX9 expression, similar to its role in triple-negative breast cancer^[33]^. However, we noted that SOX9 protein expression in U118MG cells decreased less significantly after TMZ treatment compared to other cell lines. We hypothesize that this may be due to increased protein stability influenced by the specific cellular microenvironment. For instance, recombinant therapeutic proteins in Chinese hamster ovary cells exhibit slower degradation than in other mammalian cells, largely due to differences in chaperone protein regulation^[46]^. Research on CDK7- and BRD4-mediated drug resistance in GBM cell lines (A172, U118MG, U87MG, and U251) remains limited. Nevertheless, some studies suggest that CDK7 maintains glioma stemness and confers apoptosis resistance in A172 cells by activating pro-survival pathways such as STAT3 and Notch^[47]^. U87MG and U251 cells express high levels of TFs such as MYC and STAT3, whose stability is dependent on BRD4-driven transcriptional elongation^[48]^ and CDK7-mediated phosphorylation^[49]^. Previous studies have established that CDK7 and BRD4 jointly support the SOX9-dependent SE transcriptional program, promoting aberrant cyclin expression and therapy resistance. Based on this, we explored the effect of combined treatment with the CDK7 inhibitor THZ2 and the BRD4 inhibitor JQ1 in modulating SOX9-mediated TMZ resistance in GBM. Our findings demonstrate that both THZ2 and JQ1 exhibit antitumor effects in GBM and display synergistic efficacy when combined with TMZ. We propose that THZ2 may enhance TMZ sensitivity through downregulation of SOX9 expression.

In summary, our results suggest that CDK7 inhibition represents a promising therapeutic strategy for GBM. THZ2 emerges as a potential alternative for treating chemotherapy-resistant GBM. Moreover, our data highlight the significant role of SOX9 in mediating chemoresistance. Nevertheless, several limitations remain. The precise mechanisms through which SOX9 contributes to chemoresistance in GBM - and how THZ2 reverses this process - require further investigation. Our *in vivo* experiments were limited to subcutaneous xenograft models, and validation using orthotopic models is warranted to confirm these findings. Additionally, the long-term safety and potential side effects of THZ2, alone or in combination with TMZ, must be evaluated in future studies.

## DECLARATIONS

### Availability of data and materials

All datasets and materials generated in this study can be provided by the corresponding author upon reasonable request.

### Copyright

© The Author(s) 2025
