# Supplementary material for: Super-enhancer inhibitors THZ2 and JQ1 reverse temozolomide resistance in glioblastoma by suppressing SE-driven SOX9 expression
Source: Cancer Drug Resist. 2025 Jul 22;8:37. doi: 10.20517/cdr.2025.105 (PMC12366492; doi:10.20517/cdr.2025.105)
Supplement: Supplementary file 1 [file cdr-8-37-SupplementaryMaterials.pdf]

Supplementary Materials

Super-enhancer inhibitors THZ2 and JQ1 reverse temozolomide resistance in glioblastoma by suppressing SE-driven SOX9 expression

Xinqi Teng[1,2,#], Yiming Wang[1,#], Qiang Qu[3,4,#], Weixin Xu[1], Haihui Zhuang[1], Yiwen Wei[1], Yinghuan Dai[5], Jian Qu[1,6]

[1]Department of Pharmacy, the Second Xiangya Hospital, Central South University; Institute of Clinical Pharmacy, Central South University, Changsha 410011, Hunan, China.

[2]Department of Pharmacy, Wuhan No.1 Hospital (Wuhan Integrated TCM & Western Medicine Hospital), Wuhan 430022, Hubei, China.

[3]Department of Pharmacy, Xiangya Hospital, Central South University, Changsha 410078, Hunan, China.

[4]National Clinical Research Center for Geriatric Disorders, Xiangya Hospital, Central South University, Changsha 410078, Hunan, China.

[5]Department of Pathology, the Second Xiangya Hospital, Central South University, Changsha 410011, Hunan, China.

[6]Hunan key laboratory of the research and development of novel pharmaceutical preparations, Changsha Medical University, Changsha 410219, Hunan, China.

[#]Authors contributed equally.

Correspondence to: Dr. Jian Qu, Department of Pharmacy, the Second Xiangya Hospital, Central South University; Institute of Clinical Pharmacy, Central South University, No.139 Middle Renmin Road, Changsha 410011, Hunan, China. E-mail: qujianstanley@csu.edu.cn

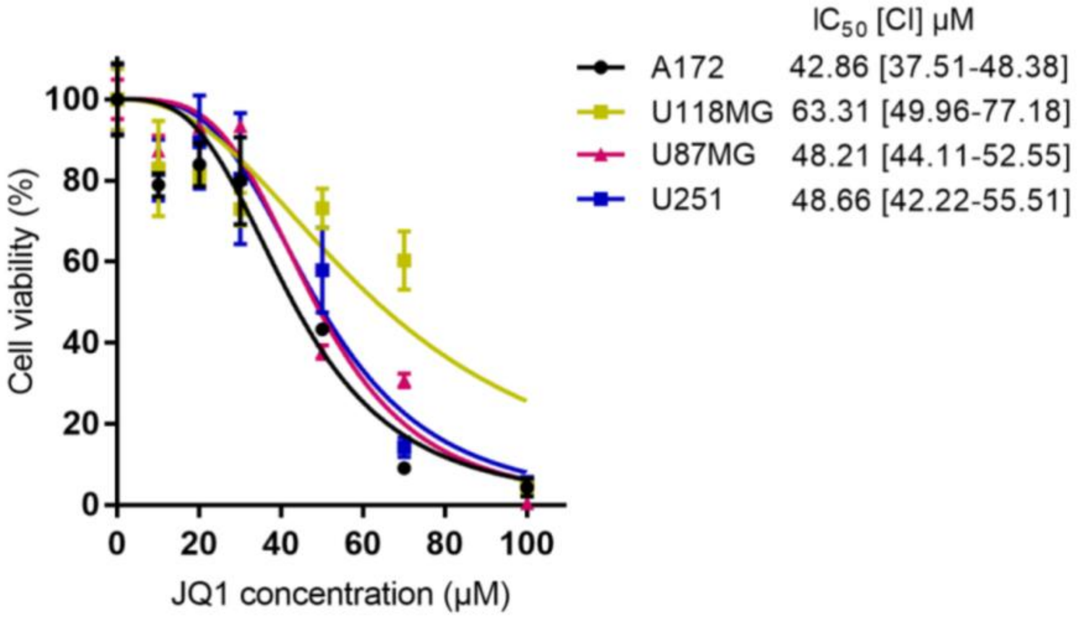

Supplementary Figure 1. JQ1 suppresses GBM cell viability. The IC_{50} of JQ1 on 4 GBM cell lines. Data represent the mean ± SD (n = 3).
